# Interrelational Proteomic Sequence Features Enhance Predictive Modeling: Application to COVID-19 Severity

**DOI:** 10.3390/biomedicines14020378

**Published:** 2026-02-06

**Authors:** Radwa El-Awadi, Oscar D. Gomez, Daniel Castillo-Secilla, Carolina Torres, Luis J. Herrera, Ignacio Rojas, Francisco M. Ortuño

**Affiliations:** 1 Department of Computer Engineering, Automation and Robotics, University of Granada, 18071 Granada, Spain; 2 CoE Data Intelligence, Fujitsu Technology Solutions S.A., 28224 Madrid, Spain; 3 Department of Biochemistry and Molecular Biology III and Immunology, University of Granada, 18071 Granada, Spain

**Keywords:** proteins, web server, protein interrelation, multiple sequence alignment (MSA), COVID-19, SARS-CoV-2, classification, feature extraction

## Abstract

**Background**: Comparing biological properties among related proteins has traditionally benefited research in areas such as biomedicine, phylogeny and evolution. Moreover, these kinds of properties have significantly increased as a result of the development of open-access resources and databases. In this context, the multiple sequence alignment (MSA) methods continue to be extensively applied to compare protein sequences and to identify evolutionarily conserved regions. **Methods**: In this work, we present INPROF, a novel web server designed to centralize and automate the computation of a large collection of features derived from protein sequences. This tool allows us to deeply analyze protein relationships and their conserved information by comparing them through their MSA. Specifically, this platform computes up to 46 different metrics including information at several proteomic levels (categories) like sequences, structures, domains or ontological terms. INPROF was designed to enable bioinformaticians and researchers to create a complete catalogue of features for subsequent prediction and artificial intelligence modeling based on proteins. The INPROF web server and source code are freely available. **Results**: INPROF were validated by predicting disease’s severity in several RNA-Seq datasets from COVID-19 patients. Specifically, INPROF were extracted from canonical proteins related to differentially expressed genes. Classification performance proved that INPROF were able to accurately classify COVID-19 severity, even outperforming classical classification with expression data. **Conclusions:** INPROF web server is a flexible platform designed to compute 46 quantitative metrics describing protein interactions which provide biologically meaningful characteristics applicable to downstream classification and prediction algorithms.

## 1. Introduction

The study of the relationships among genes or proteins has traditionally been considered a key task in understanding the underlying biological processes. However, it is still under discussion what the most adequate properties are to describe and quantify such relationships [1]. Consequently, several studies have recently proposed different measurements for this purpose [2]. These metrics are often calculated based on different biological properties: from the structural level (primary to quaternary) to functional level (domains, ontologies, etc.). For example, ATLAS [3], SoluProt [4], GTalign [5], ShiftCrypt [6], ProteoVision [7], or MAFFT-DASH [8], and the more recent EFG-CS [9] are web servers that leverage protein sequence and structural information to compute quantitative descriptors. Similarly, several tools implement approaches that integrate heterogeneous data at the protein structural level including PASSer [10], IntFOLD [11], SWISS-MODEL [12] and the latest developments of AlphaFold 3 [13], which provide highly accurate structural predictions and further incorporate the ability to model protein interactions with ligands (small molecules, DNA/RNA, and other proteins). Furthermore, newly developed platforms such as ProFeatX [14] and ESMFold [15] provide advanced structure prediction capabilities using deep learning and protein language models. Finally, several tools focus on functional-level properties, including g:Profiler [16], as well as recent updates such as ShinyGO [17] and Metascape [18], which integrate pathway enrichment and interpretation of protein interaction.

Regardless of the biological level considered, sequence conservation is a key indicator of protein interrelationships [19]. Thus, evolutionary conservation in biological sequences is commonly analyzed using pairwise alignment strategies. Moreover, these alignments need to be extended when three or more sequences are contrasted by applying multiple sequence alignment (MSA) approaches [20,21]. In this sense, MSAs are of great relevance in determining sequence similarities and can further complement information extracted from other biological levels, such as protein structure or functional annotations. For example, recent studies have successfully applied MSA tools to predict evolutionary relationships [22], phylogenetic trees [23,24], or infer protein–protein interactions [25,26]. Importantly, beyond their classical use for evolutionary inference, MSAs can also be considered as quantitative tools to summarize sequence-level similarity, heterogeneity, and structural compatibility within sets of proteins, even when they are less evolutionarily related. Consequently, the application of MSAs has proven to be extremely valuable for downstream analysis, including domain identification [21], structure prediction [13], and functional inference. In fact, recent studies have shown that incorporating information captured by MSAs can substantially enhance RNA- and protein-level modeling, motivating integrative approaches that bridge transcriptomic data with protein sequence, structural, and functional features [20].

In light of the interest of these features for the assessment of protein and gene relationships, we present a new web-based platform, named INterrelational PROtein Features (INPROF). Although simplistic, this web server allows users to select multiple similarity measures based on several protein properties. Specifically, users can extract over 46 relationship features among several sets of proteins. Consequently, every user can create their own customized protein-feature datasets for downstream analysis purposes. Several metrics computed by INPROF are directly derived from MSA results, as alignments are considered essential for the understanding of the relationship among proteins. In this sense, the exact feature dataset produced by this web server was already considered in subsequent bioinformatics machine learning tools related to sequences and MSA [27,28]. Moreover, some related feature subsets have been built based on the protein relevant knowledge for other recent machine learning studies. For example, similar protein features were found to be significantly important for predictions such as protein–protein interactions (PPIs) [29], evolutionary phylogenetic trees [30] or prediction of substrate specialties in membrane transport proteins [31] or palmitoylation sites [32]. More recently, deep learning models have reinforced the importance of integrating structural and evolutionary protein features for accurate predictive analysis [26]. However, to the best of our knowledge, no existing tool integrates such a simple and comprehensive collection of interrelated sequence quantitative features.

Finally, we conducted a real-world case study as a proof of concept illustrating how INPROF-derived features can be applied to explore disease severity patterns among COVID-19 patients. Since March 2020, SARS-CoV-2 has shaken life, and several studies have been developed to discover how the virus works. This virus is characterized by the heterogeneity of its symptoms, depending on the patients’ conditions [33]. Although most cases are asymptomatic or mild [34], there are COVID-19 patients who develop severe pulmonary symptoms [35] such as acute respiratory distress syndrome (ARDS), pulmonary edema, intense kidney injury and multiorgan failure, even leading to death [35,36]. Several studies have shown that Differentially Expression Genes (DEGs) are useful for studying the disease and its symptoms. More specifically, prior research has reported links between COVID-19 patient gene expression and the specific disease, investigating how changes in gene expression influence COVID-19 outcomes [37,38,39]. In this study, we show that INPROF-derived features can be applied to severity classification using proteins associated with DEGs, achieving similar performance and providing complementary predictive information compared to models based solely on DEGs.

## 2. Materials and Methods

### 2.1. Implementation and Usage

The INPROF web server can be directly consulted online without any installation requirement (https://www.ugr.es/~fortuno/inprof/inprof.php, accessed on 22 January 2026). Users can interact through the designed web server using a simple and intuitive web interface. Additionally, features can also be extracted from the web server through an API by using standard programming languages like Python (v3.12.3), Perl (v5.34.0) or R (v4.5.1). An example of a request using a Python script is available on the website. INPROF requires as input a set of at least two proteins, each unambiguously identified by its UniProtKB accession or name, in order to compute interrelational features.

**Figure 1:**
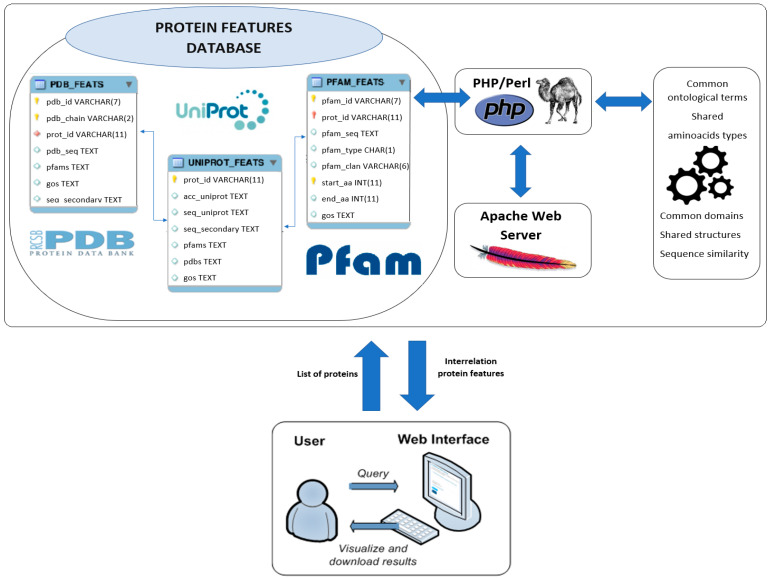
INPROF Web Server scheme is composed of: (i) Web Interface; (ii) Apache/PHP Web Server; (iii) Protein Feature Calculation (Perl module); (iv) Protein Feature Database.

This web server is made up of four well-defined software modules (see Figure 1): web interface, Apache web server, protein feature calculation, and local database of protein features. Each of those components, together with a detailed description of the features retrieved, is presented in detail in the following subsections.

#### 2.1.1. Protein Features

The protein features computed by INPROF are classified into different protein categories (see complete list of features in Supplementary Table S1). Each category refers to the group of metrics that users can calculate separately to assess the relationships between proteins. These feature categories are:

- **Sequences:** Basic statistical properties of amino acid chains, such as average length or length variance. For MSA, this category performs statistics about matches in the alignment.
- **Amino-acid Types:** Percentages of different chemical classes of amino acids in the sequence (e.g., uncharged, nonpolar aliphatic, positively charged, etc.) composing the chain.
- **Domains:** Properties related to functional domains as defined by Pfam [40]. Both Pfam domains and their corresponding clans are considered.
- **Secondary Structure:** Percentage of amino acids located in different protein’s secondary structures (*α*-helix, *β*-strand, hydrogen-bonded turn). This information is retrieved from Uniprot [41].
- **Tertiary Structure:** Properties regarding annotated structural data according to the Protein Data Bank (PDB) [42]. Among others, structural contacts are computed here. A contact is considered when any pair of atoms in two different amino acids, separated by more than five residues in the sequence, are close enough that no solvent molecule can occupy the space between them (similarly defined in [43]).
- **Ontological Terms:** Features related to shared terms in the three ontologies defined by Gene Ontology (GO) [44]: biological process (BP), molecular function (MF) or cellular component (CC). Note that metrics in this category cannot be referred to MSA. Since GO terms are not associated to a specific location along sequences, alignment metrics cannot be computed from them.

When selected by the user, these features can be optionally expanded using multiple sequence alignment (MSA) among proteins. In this case, the resulting metrics are reinterpreted based on both the protein sequences and their alignments. These MSA-based features are not explicitly intended to infer evolutionary relationships among proteins, but rather to quantify complementary descriptors (sequence-level similarity, heterogeneity, and alignment coherence) within a group of proteins enriching protein representations for downstream classification tasks. Additionally, most INPROF are computed as normalized or aggregated statistics to ensure comparability across protein sets of different sizes. However, the total number of input proteins is still included as a separate feature to explicitly capture potential biological differences that may be associated with it.

Although INPROF requires valid UniProtKB identifiers, custom/partial protein sequences may be provided by users associated to those identifiers, not necessarily using those sequences already available in UniProtKB. In this case, all sequence- and alignment-based features as well as annotations related to those (domains, structures, etc.) are computed based on the overlapping with the supplied sequences. Table 1 summarizes the number of features included in each category. A detailed description of each individual feature and its computational procedure is provided in the tool’s documentation (https://www.ugr.es/~fortuno/inprof/help.html, accessed on 22 January 2026).

#### 2.1.2. Web Interface

Protein queries can be provided directly by users specifying their Uniprot entry names or accession numbers [41]. They can be delivered both directly from a textbox at the web interface or uploaded as a list in a text file. Alternatively, protein sequences can be imported from a FASTA format file. In that case, sequences in the file are considered preferential for the computation, instead of downloading sequences from Uniprot.

Users can interactively select the desired feature categories (see above) through multiple checkboxes in the web interface. Moreover, if desired, they may choose to apply an MSA approach to obtain an extended set of features (see Table 1). In this case, three alignment tools are available: CLustalW [45], T-Coffee [46], and MUSCLE [47]. Although these tools represent classical approaches, they remain the most widely used MSA tools in current bioinformatics workflows. Finally, the computed similarity measures are provided in a downloadable tabular format, whereas the resulting sequence alignments can also be exported in MSF format if this option is selected.

In addition, the web interface enhances the feature table by linking each metric to its corresponding external resource. This allows users to directly access the original data sources from which each metric was derived, including databases such as UniProt, Pfam, PDB, and Gene Ontology, as previously described.

#### 2.1.3. Apache Web Server

The back-end web server runs through a PHP module by managing the user query and generating the output results. This module includes SOAP-based web services, which can be queried from standard RESTful POST functions. INPROF are then internally processed through an in-house Perl script based on user preferences. After features have been computed, a response in JSON format is served to the front-end (or the querying user) with every requested feature, including their identifiers (labels), their metric values, and a list of complementary identifiers from external resources.

#### 2.1.4. Metrics Calculation (Perl Module)

The 46 features are computed according to the categories and options selected by the user. Feature calculation is carried out in the back-end module by querying annotation data from a local MySQL database (see below for details). When a list of proteins is submitted, two scenarios may occur. If all proteins are already stored in the local database, the requested metrics are immediately computed, and the results are returned to the user. If some proteins are missing, the system automatically updates the local database by retrieving the missing information from external sources, which may temporarily increase the response time. To minimize such delays, the database is periodically updated and maintained to ensure complete protein coverage and quick query processing. Once the database has been refreshed, all features are computed consistently using newly retrieved protein annotations.

#### 2.1.5. Protein Feature Database

The INPROF database is built in a MySQL framework with three main tables, each one connected to a different external source. The idea behind this layout is to combine several biological sources while keeping the structure manageable and consistent. A brief description of these tables is given below:

- **UniProt_Feats:** This table includes all annotation details retrieved from Uniprot [41]. It also serves as the central table, linking each protein to its corresponding entries in the Pfam and PDB tables, which helps integrate both structural and functional information.
- **Pfam_Feats:** This table contains information on functional domain obtained from the Pfam database [40]. It includes domain names, their positions along the protein sequence, and their associated families or clans.
- **PDB_Feats:** This table stores the tertiary structure information provided by the Protein Data Bank [42], such as chain identifiers, the sequence regions to which they map, and several additional descriptors commonly used to describe protein structure.

The database is periodically updated with the latest external annotations. At the moment, it includes information for more than 560,000 proteins. Anyway, when a user requests a new protein with a valid UniProtKB accession which is not yet available locally, the system retrieves the necessary annotations in real time and incorporates them into the local database, to guarantee an optimal response time.

### 2.2. Case Study: COVID-19 Severity Classification

**Figure 2:**
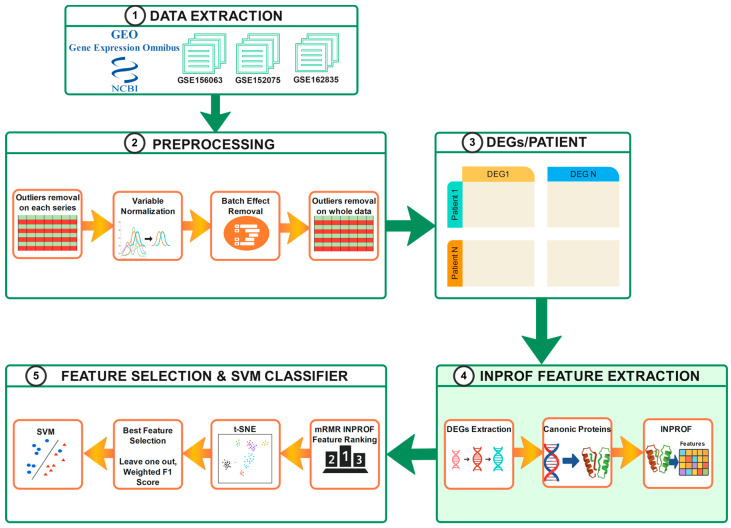
INPROF pipeline for COVID-19 severity classification. The workflow includes: (**1**) data extraction from GEO; (**2**) preprocessing with outlier removal, normalization, and batch effect correction; (**3**) patient-level DEGs computation; (**4**) INPROF feature extraction (highlighted in green as the main methodological contribution), where DEGs are mapped to canonical proteins to generate INPROF; and (**5**) feature selection and SVM classification using mRMR ranking, t-SNE visualization, and LOOCV.

To evaluate the functionality of INPROF, we analyzed publicly available RNA-Seq datasets from patients infected with COVID-19. Specifically, we reproduced the severity classification study presented in [39]. In that study, the authors used the same GEO datasets and the severity classification relied exclusively on differential gene expression values. By applying INPROF to the same patient groups, we ensured that both approaches were evaluated under comparable conditions. Our objective was to assess whether the protein interrelated features created by INPROF can serve as an alternative and complement to gene expression models for severity classification. The complete validation workflow is summarized in Figure 2. All code used to reproduce the study, including data preprocessing, INPROF feature extraction, and model training, is available in our GitHub (git v2.43.0) repository: https://github.com/fortuno/INPROF/tree/main/INPROF-COVID19-Severity, accessed on 22 January 2026.

#### 2.2.1. Data Acquisition

The datasets were obtained from the NCBI Gene Expression Omnibus (GEO) [48], corresponding to the series GSE156063 [49], GSE152075 [50] and GSE162835 [51]. Data was initially retrieved as raw count matrices. Severity metadata was explicitly available only for GSE162835; for the remaining series, clinical labels were retrieved by contacting the authors of the datasets via email.

The patients in these series were labeled into four classes: *Outpatient* (asymptomatic or mild cases), *Inpatient* (hospitalized, non-ICU), *ICU* (intensive-care patients) and *Control*. In this study, the *Control* group is used as the reference baseline, comparing each *Outpatient*, *Inpatient*, and *ICU* sample with all *Control* samples to compute the metrics of expressed genes in terms of log2 fold-change and *p*-value. After a careful preprocessing and outliers removal with the R-package KnowSeq [52] (see details below), the resulting cohort comprised 206 patients: 131 *Outpatients*, 18 *Inpatients*, 10 *ICUs*, and 47 *Controls*, each with expression profiles of 15,526 genes.

#### 2.2.2. Differential Expression Analysis

The gene expression matrix for each patient group (*Control*, *Outpatient*, *Inpatient*, *ICU*) was built using KnowSeq package [52]. KnowSeq comprises all standard steps for transcriptomic gene expression analysis including mapping, preprocessing, outlier removal, normalization or differential expression analysis (underlaying standard packages like limma, cqn or edgeR are applied). Since original data was obtained as raw counts, outliers were initially removed using both an inter-quartile range (IQR) technique and MA-plot. MA-plot were represented by calculating Hoeffding’s statistic on the joint distribution of A and M for each of the samples [53]. Later, the batch effect, which is an intrinsic deviation of data produced on its extraction, was removed through the Surrogate Variable Analysis (SVA) method [54].

After preprocessing and normalization, differentially expressed genes (DEGs) were individually identified for each COVID-19 patient group (*Outpatient*, *Inpatient* and *ICU*) compared to the *Control* group. A refined subset of DEGs was obtained for each patient applying the Benjamini–Hochberg correction to control the False Discovery Rate (FDR) (adjusted p-value < 0.001) and imposing a threshold on the log_2_ fold-change (LFC > 1.5). To limit the computational load during INPROF feature generation, a maximum of 170 top-ranked DEGs per patient were retained.

#### 2.2.3. Retrieval of Canonical Proteins

The objective of this step is to generate a list of proteins associated with the DEGs identified for each COVID-19 patient. Once DEG lists were obtained, the corresponding protein products were determined by mapping each gene to its encoded protein. The canonical protein, defined as the amino acid sequence most likely to be expressed, was selected for further analysis.

The list of DEGs was first obtained using their gene names and subsequently annotated with their respective *Ensembl* identifiers [55]. Once this mapping was completed, the canonical protein sequences were obtained through the *UniProtID* mapping service. In this way, each patient could be represented by a defined set of proteins associated with their specific DEG profile.

#### 2.2.4. INPROF Feature Extraction

The resulting protein datasets were then analyzed using INPROF for feature extraction. For the protein list of each patient, INPROF computed the 46 features describing both individual protein characteristics and properties derived from sequence alignments. In this study, MUSCLE was selected as the alignment tool; however, comparable results were obtained with the alternative options, ClustalW and T-Coffee. From the initial INPROF dataset, two alignment-related features (*MSA_TC* and *MSA_TN*) were excluded, as they yielded zero values across all samples and therefore did not provide discriminatory information. Consequently, the final dataset used for the analysis contained 44 informative features for the 159 patients with COVID-19 (the entire INPROF-based feature dataset is provided in Supplementary Table S2).

#### 2.2.5. Feature Selection

After retrieving the final feature dataset, several machine learning algorithms were applied to evaluate the ability of these features to predict the severity of COVID-19. Initially, a preliminary graphical perspective about patient’s distribution on a bi-dimensional space was performed by two complementary dimensionality reduction approaches: t-SNE (t-Distributed Stochastic Neighbor Embedding) [56] and UMAP [57]. These models were performed with the R language Rtsne [58] and umap [57], respectively.

Nevertheless, increasing the complexity of the predictive models is expected to substantially enhance the separation observed in the t-SNE representation. To this end, the minimum Redundancy Maximum Relevance (mRMR) algorithm [59] was applied using the *FeatureSelection* function from KnowSeq. This approach generates a ranked list of features based on their relevance and redundancy. Thus, subsets of increasing size were progressively evaluated to identify the optimal feature combination for classification.

#### 2.2.6. Classification Model

Although several classical classification models (KNN, Random Forest and SVM) were assessed, SVM yielded the best overall performance for this task. To determine the minimal number of features required to accurately discriminate among patient groups, a separate SVM classifier was trained for each progressively expanded subset of features ordered according to the previous mRMR ranking. Because our INPROF-derived model was compared against a DEG-based approach applied to the same datasets [39], we adopted an equivalent training and feature selection strategy to ensure comparability under identical conditions. Briefly, each model was trained using an 80/20 train–test split, followed by a 5-fold cross-validation on the training set to determine both the optimal number of features and the best performing hyperparameter configuration. Every model was evaluated using standard classification metrics: accuracy, sensitivity, specificity and F1-score.

## 3. Results

**Figure 3:**
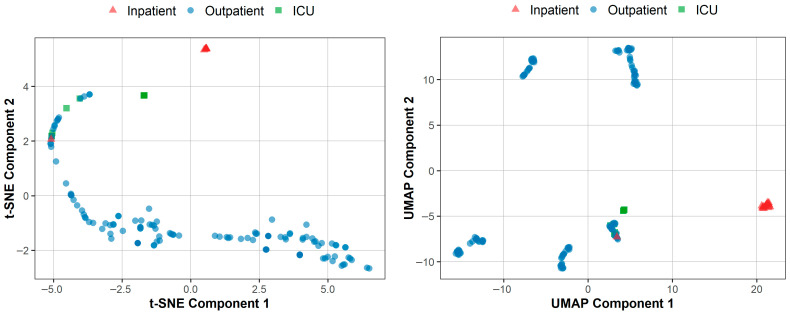
Dimensionality reduction algorithms t-SNE (**left**) and UMAP (**right**) applied to 44 INPROF for COVID-severity dataset.

The previously described case study was conducted to assess the utility of the INPROF web server and its interrelated proteomic features. Initially, a subset of proteins was obtained for each patient from the list of DEGs obtained comparing against *Control* (see Section 2.2 for details). INPROF were then calculated for the list of protein of each patient. Features were initially reduced to a bi-dimensional space by both t-SNE and UMAP approaches, for an easier visualization purpose (Figure 3).

As shown in t-SNE (Figure 3, right panel), *Inpatient* and *ICU* groups are mostly clustered toward more distinct regions of the map, while the *Outpatient* samples form a broader and more dispersed distribution. Although the t-SNE representation may visually suggest a gradual separation among severity groups, such patterns should be interpreted with caution, as t-SNE may introduce noisy and artificial gradients not reflecting true biological trajectories [60]. To provide a complementary and more robust visual assessment, we additionally applied UMAP dimensionality reduction, which may preserve global structure better, representing more clinically meaningful variation. As shown in the UMAP projection (Figure 3, left panel), the three severity groups still form clearly separated clusters, reinforcing the notion that INPROF-derived features capture differences associated with COVID-19 severity. Notably, the *Outpatient* samples are further subdivided into multiple subclusters in UMAP, suggesting the presence of latent heterogeneity within this group that may be related to sample date, host response, comorbidities, or technical biases (for details, see limitations in Section 5). Although both visualizations indicate an acceptable separation among mild, moderate, and severe patient profiles, more sophisticated modeling approaches are required to capture complex non-linear patterns which better classify samples that are ambiguously positioned near incorrect classes

In order to improve the separation of the severity groups, a more complex feature ranking based on the mRMR algorithm was then performed. Although 44 INPROF were initially retrieved, those exhibiting very low variability across samples (specifically, features with fewer than 10 non-zero values) were excluded from this ranking to prevent training models on features with no discriminatory information. More specifically, a total of six INPROF, primarily related to 3D structural similarity and Pfam alignment metrics, were excluded from the ranking: the number of shared 3D structures (*SEQ_CS*), the number of structural contacts (*SEQ_NC*), the number of contacts correctly aligned (*MSA_3D*), the STRIKE score for alignment structural accuracy (*MSA_SK*) and the percentage of matches in the same domains (*MSA_PT*) and the same clans (*MSA_PC*). These metrics were found less informative for the heterogeneous protein sets derived from COVID-19 severity-associated DEGs, given their lower degree of functional and structural similarity. Nevertheless, the discarded features were found to be ranked near the bottom of the ranking. The final mRMR ranking was then obtained for the remaining highly variable 38 INPROF.

**Figure 4:**
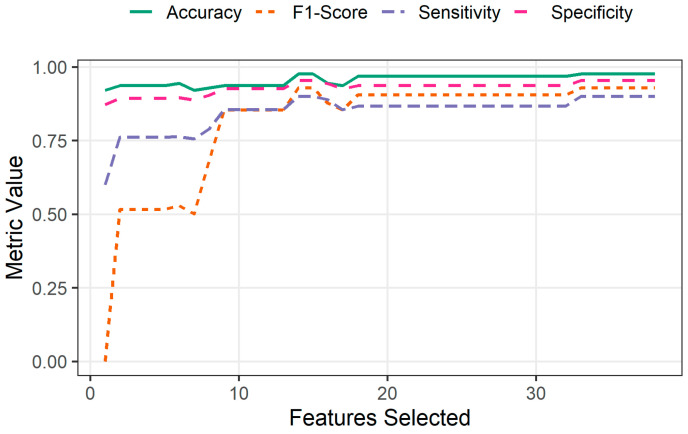
Evolution of individual SVM classifier performance across progressively selected features with mRMR using 5-fold cross-validation in training subset. Four classical classification performance metrics (Accuracy, F1-Score, Sensitivity, and Specificity) are shown.

After ordering the INPROF feature, several SVM classification models were performed. Specifically, a total of 38 SVM models were constructed (one per feature ranked by mRMR), where the *n*-th model incorporated the top *n*-ranked features. As shown in Figure 4, the performance of the SVM classifier in the training subset improves rapidly as the first features are incorporated, stabilizing around approximately 10 features. Beyond this point, all metrics exhibit only minor fluctuations, indicating that most discriminative information is captured within the top-ranked features.

Detailed metric values for selected subsets (2, 8, 10, and 14 features) are reported in Table 2, providing a quantitative summary of the classifier’s behavior at key stages of the feature selection process. As shown in the table, classification performance generally increases with the number of features used for model training, accompanied by a clear reduction in standard deviation across the 5-fold cross-validation. This decreasing variability indicates that models become more stable and robust as additional informative INPROF are incorporated. Although the test subset performance is consistently lower than training performance, the results remain strong overall. Most of this reduction, particularly in Sensitivity and F1-score metrics, was anticipated due to the pronounced class imbalance, which makes correctly identifying the minority class (*ICU* samples) substantially more challenging. For this reason, and to remain consistent with the DEG-based study used for comparison [39], a leave-one-out cross-validation (LOOCV) scheme was additionally applied for a selected number of features to enhance the statistical robustness of the evaluation, given the limited number of *ICU* samples available. Specifically, as already observed in Figure 4, we considered the top-10 INPROF, as they provide a sufficient balance between high accuracy and overall performance across metrics. These features, which were found to be meaningful in explaining differences between COVID-19 patients with varying severity grades, are briefly presented here in the same order as they were ranked by mRMR:

1. Percentage of polar uncharged amino acids in protein sequences (*SEQ_PL*).
2. Number of Pfam clans shared between each protein pair (*SEQ_CK*).
3. Variance of the length in the protein sequences (*SEQ_VA*).
4. Maximum length in the protein sequences (*SEQ_MX*).
5. Number of Pfam domains shared between each two proteins (*SEQ_CT*).
6. Percentage of amino acids in protein sequences with atypical or unknown secondary structures (*SEQ_SU*).
7. Minimum length in protein sequences (*SEQ_MN*).
8. Percentage of amino acids in protein sequences that are included in any Pfam clan (*SEQ_PC*).
9. Percentage of matches in the alignment between pairs of amino acids included in β-strand secondary structures (*MSA_TD*).
10. Percentage of gaps in the alignment. This is a measure of how similar the protein sequences are (*MSA_GP*).

**Figure 5:**
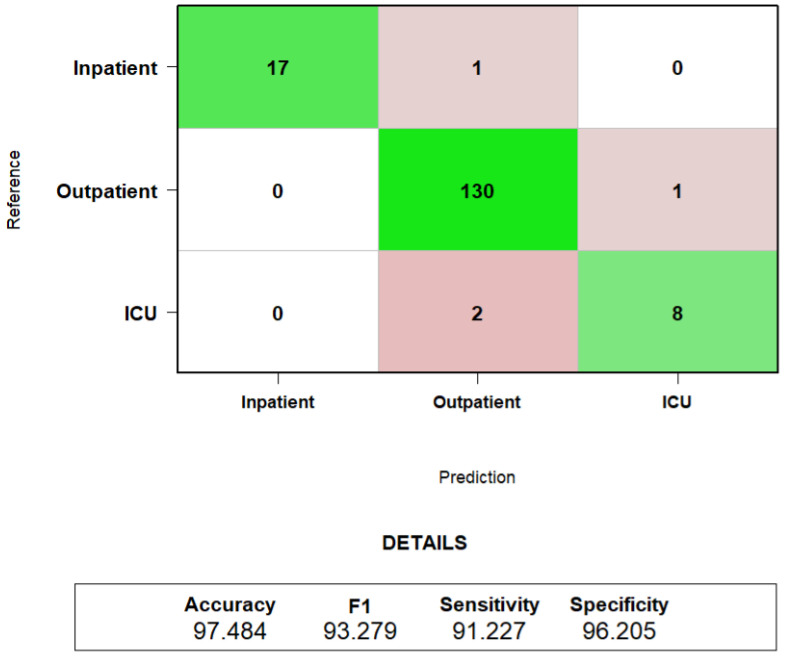
Confusion matrix and performance metrics (Accuracy, F1-Score, Sensitivity and Specificity) obtained with SVM and the top 10 ranked features applying a LOOCV evaluation scheme.

The confusion matrix obtained and its corresponding performance metrics applying the LOOCV evaluation scheme over the top-10 selected features is shown in Figure 5. The model achieves an overall accuracy of 97.48%, with strong performance across all three groups. *Inpatient* and *Outpatient* cases are classified with high reliability, with only one patient misclassified per group. Furthermore, despite the limited number of *ICU* samples, the model correctly identifies most of those patients (8 out of 10), reflecting balanced and robust behavior. The corresponding performance metrics (F1-score: 93.28%, Sensitivity: 91.23%, Specificity: 96.20%) indicate that the model is able to generalize reliably even though the validation setup was intentionally strict.

As mentioned earlier, we compared the INPROF-based model with the DEG approach published by Bajo et al. [39]. To match the structure of our analysis, the *Control* group was removed from their confusion matrix, keeping the three classes of clinical severity: *Outpatient*, *Inpatient*, and *ICU*. It is worth noting that the number of samples in each category differs slightly between the two studies due to variations in preprocessing and outlier removal. After comparing under the same evaluation conditions, INPROF model achieved slightly higher overall performance than the DEG model, with modest improvements in accuracy (97.5% vs. 96.8%) as well as in F1-score, sensitivity, and specificity. The most noticeable difference was observed in the *Outpatient* group, where the DEG model misclassified three samples to the *ICU* class. In contrast, both models correctly identified most of the *ICU* samples, even when they are the smallest and most challenging group. Collectively, these findings suggest that the proteomic interaction features generated by INPROF provide a robust and discriminative representation of patient molecular profiles. These results can offer a reliable complement to DEG-based markers with a comparable performance.

## 4. Discussion

The main purpose of this study was to introduce the INPROF web server and to assess whether it can produce biologically informative features based on protein interactions. To address this goal, we built a unified analysis pipeline that combined several publicly accessible and heterogeneous COVID-19 RNA-Seq datasets. For each patient, we identified differentially expressed genes (DEGs) and mapped them to their respective canonical proteins. This step enabled the extraction of interaction-level features through INPROF, which were subsequently used for disease severity classification. This analysis allowed us to explore the discriminative potential of these features and to assess whether INPROF captures patterns relevant to clinical outcomes for this specific case study.

The findings of our analysis suggest that the features generated by INPROF yield a robust and biologically coherent representation of the molecular states of the patients. Specifically, classification performance improved progressively as more features were incorporated, reflecting both an increasing accuracy and a reduction in variability across cross-validation folds. This pattern shows that these interaction-driven proteomic features may capture complementary biological signals distributed across multiple proteins, rather than being constrained to the influence of individual genes. Interestingly, once the model incorporated around ten features, its performance largely stabilized, indicating that only a small number of interaction-based measures are sufficient to reliably separate severity groups. This observation is consistent with the broader view that the severity of COVID-19 arises from coordinated molecular disruptions rather than changes confined to isolated genes.

**Figure 6:**
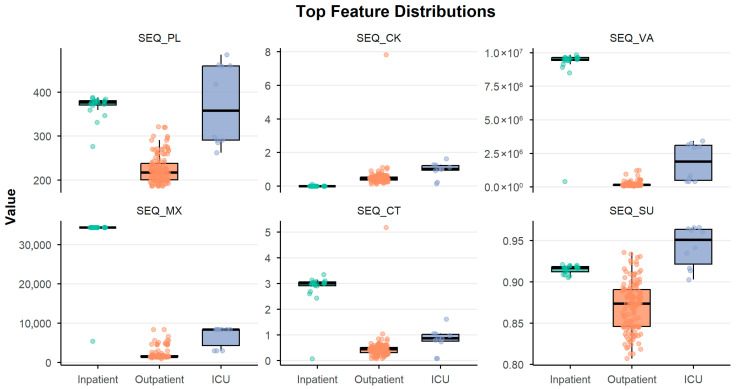
Distribution of the top six INPROF-derived features distinguishing COVID-19 severity groups. Boxplots show the variation in the six most informative features (*SEQ_PL*, *SEQ_CK*, *SEQ_VA*, *SEQ_MX*, *SEQ_CT* and *SEQ_SU*) across the three clinical categories (*Outpatient*, *Inpatient* and *ICU*).

The group of ten features ranked by the mRMR procedure provides interesting insights about the molecular attributes most closely related to the severity of disease. Several of the top-ranked features captured differences in protein sequence length (*SEQ_VA*, *SEQ_MX*, *SEQ_MN*). Interestingly, higher length variances were usually found in proteins from Inpatient and ICU patients (see *SEQ_VA* in Figure 6), suggesting that the diversity of protein size may indicate functional heterogeneity associated with severe disease. In fact, variation in protein length often reflects changes in domain architecture or structural modularity, influencing functions such as inflammation and antiviral activity [61]. This observation aligns with other analyses of the SARS-CoV-2 interactome, which show that many host proteins involved in viral entry, replication, and immune control strongly depend on their structural configuration and the way they interact with other molecules [62].

Consistent with this interpretation, other top-ranked features were also associated with Pfam domains (*SEQ_CK*, *SEQ_CT*, *SEQ_PC*), indicating that conserved functions play an important role in distinguishing between severity groups [63]. Moreover, recent studies suggest that many host proteins interacting with SARS-CoV-2 share common Pfam domains, reinforcing the relevance of domain-level organization in viral–host interactions and its possible influence on clinical outcomes [64]. Within our dataset, proteins from *Inpatient* and *ICU* patients repeatedly showed a higher number of shared domains compared to those from *Outpatients* (see *SEQ_CT* in Figure 6). Specifically, two domains were more frequently shared among proteins from severe patients: the *fibronectin type III (FN3) domain* (PF00041) and the *Immunoglobulin I-set domain* (PF07679). FN3 domains have been associated with cytokine binding and immune-modulatory roles [65], while *Immunoglobulin I-set* domains contribute to cell adhesion, cell–cell recognition, and immune receptor functions. All these observations support the broader understanding that severe COVID-19 is strongly influenced by dysregulated immune pathways, including exaggerated cytokine activity [66]. The entire list of shared domains in the three classes is provided in the Supplementary Table S3.

Additional informative features included metrics based on amino acid composition, such as the proportion of uncharged polar residues (*SEQ_PL*) and residues linked to unusual or undefined secondary structure annotations (*SEQ_SU*). Proteins with high sequence disorder or atypical residue composition often create flexible binding regions and support dynamic interaction behavior, a pattern well-documented in structural biology [67]. This type of conformational flexibility is increasingly viewed as an important factor in host responses to viral infection, shaping immune signaling pathways and cellular stress responses [68]. Taken together, these findings show that these INPROF again capture biologically coherent patterns linked to well-established mechanisms of COVID-19 severity, such as immune modulation, inflammation, and cellular stress responses. The fact that these features remain consistent across validation folds and datasets shows that INPROF can detect stable disease-relevant signals despite heterogeneity in transcriptomic profiles.

On the other side, INPROF derived from 3D annotations in PDB generally showed limited variability and low discriminative power in the COVID-19 severity case study. In fact, four of these features were excluded before feature selection (see Section 3) whereas the remaining two were ranked under the least informative features. This behaviour likely reflects weak structural and evolutionary relationships among DEG-derived protein sets, with rare common structures or structural similarities. Importantly, this does not imply these features are generally uninformative, but they are expected to increase their importance when more evolutionarily related or structurally conserved protein sets are considered. 

Finally, it is important to acknowledge that the INPROF tool was inherently designed as disease-agnostic and generalizable to other diseases and independent cohorts, beyond the presented COVID-19 severity use case presented here. In fact, the COVID-19 analysis presented here should be regarded as a proof-of-concept case study rather than a comprehensive benchmark. Since INPROF are derived from fundamental protein properties (sequence composition, domain organization, structural annotations or protein similarity, they are not tied to a particular pathology, but rather reflect system-level characteristics of protein sets associated with a given condition. Therefore, INPROF can be widely applied to protein lists derived from differential expression, co-expression modules, or mutational analyses in diverse contexts, including cancer subtyping and staging, autoimmune disorders, neurodegenerative diseases, and other infectious diseases. Further evaluations across independent cohorts, diseases, and classification tasks are being performed to more extensively validate the general utility and performance of INPROF-derived features. Moreover, the use of interaction-level and set-based descriptors makes INPROF particularly well suited for cross-cohort analyses, where individual gene signatures may vary but functional patterns remain conserved. These properties make INPROF a versatile tool for proteomics-driven machine learning applications in translational and precision medicine.

Nonetheless, some limitations should be considered when interpreting our case study results. The datasets were collected in different countries and during different phases of the pandemic, which can differ in the circulating SARS-CoV-2 variant at those locations and dates. Such variability can substantially influence transcriptomic patterns in ways not explicitly captured by our pipeline. Another consideration involves the way clinical labels were assigned. This assignment was based on the patient’s condition at the time of the PCR test, without information on the disease progression afterward. As suggested by the t-SNE plot (Figure 3), some individuals labeled as *Outpatient* might have later developed more severe symptoms, which could introduce some bias. Also, contextual factors, including hospital capacity, admission policies, and demographic considerations, may have affected decisions about hospital or ICU admission. These decisions may not always reflect biological severity and can introduce additional uncertainty in the reference labels. Despite that, the results were consistent across the datasets and validation schemes. The reproducibility of the selected features and the robust classification outcomes suggest that these confounders had only a limited effect on the main conclusions, reinforcing the reliability of INPROF-based representations in this specific use case.

## 5. Conclusions

This study presents the INPROF web server, a flexible platform designed to compute quantitative metrics based on interactions that describe how proteins relate to each different biological dimension. INPROF integrates information from several sources, including protein sequences, amino acid classifications, Pfam domains, and structural attributes such as secondary and tertiary structures, together with annotation descriptors. Because INPROF relies on these external annotation resources, proteins must be correctly identified in UniProtKB to enable feature computation, which may restrict its use for unidentified or insufficiently annotated protein sequences. When required, the platform can also incorporate additional metrics derived from multiple sequence alignments (MSAs) computed for these same biological properties. In total, INPROF provides up to 46 distinct metrics, enabling a comprehensive and multidimensional characterization of protein similarity. Unlike approaches that focus primarily on enrichment analysis or functional annotation, the key strength of INPROF lies in its ability to produce broad quantitative features describing how proteins relate to each other in a reliable and biologically meaningful manner. Thus, these features can be used directly for downstream applications, including classification, clustering, and incorporation into machine learning workflows.

As a specific use case, INPROF was applied to a COVID-19 RNA-Seq dataset, demonstrating that these proteomic features accurately predict disease severity. This use case validated both the functionality and the biological relevance of the platform. INPROF can therefore be categorized as a general-purpose tool for generating complete, informative feature datasets derived from groups of proteins, suitable for diverse classification, prediction or machine learning applications. Its flexibility and quantitative nature make it a valuable resource for studies aiming to integrate protein-level information into computational analyses.

## Figures and Tables

**Table 1:** Summary of INPROF by categories. A full descriptive list is provided in Supplementary Table S1.

Feature Category	Sequence-Based	MSA-Based	Total
Sequence Statistics	5	3	8
Amino Acid Type	5	5	10
Functional Domains	6	2	8
Secondary Structure	4	5	9
Tertiary Structure	4	2	6
Ontological Terms	5	0	5
**Total**	29	17	46

**Table 2:** Average classification performance obtained using the 80% training split with 5-fold cross-validation, evaluated for selected numbers of top-ranked features. Values represent mean ± standard deviation (across folds in training).

**Training (80%)—5-Fold CV**				
**#**	**Accuracy**	**Sensitivity**	**Specificity**	**F1-Score**
2	0.937 ± 0.021	0.761 ± 0.181	0.893 ± 0.085	0.515 ± 0.471
8	0.929 ± 0.052	0.789 ± 0.168	0.904 ± 0.073	0.677 ± 0.383
10	0.936 ± 0.054	0.855 ± 0.049	0.926 ± 0.029	0.854 ± 0.060
14	0.976 ± 0.022	0.900 ± 0.091	0.954 ± 0.050	0.928 ± 0.065
**Test (20%)**				
**#**	**Accuracy**	**Sensitivity**	**Specificity**	**F1-Score**
2	0.910	0.654	0.878	0.648
8	0.879	0.642	0.867	0.642
10	0.879	0.642	0.867	0.642
14	0.910	0.654	0.878	0.648

## Data Availability

The original contributions presented in this study are included in the article/Supplementary Materials. Additionally, source codes and original raw data is available in the repository https://github.com/fortuno/INPROF (accessed on 22 January 2026). Further inquiries can be directed to the corresponding author.

## Supplementary Materials

The following supporting information can be downloaded at: https://www.mdpi.com/article/10.3390/biomedicines14020378/s1. Table S1: List of features computed by INPROF web server; Table S2: Matrix with the entire dataset retrieved from INPROF; Table S3: Top-50 Pfam domains found shared by each paired proteins.
